# Factors Influencing the Development of Metachronous Fractures in Patients with Osteoporotic Vertebral Fractures Treated with Conservative Management or Vertebroplasty

**DOI:** 10.3390/diagnostics15020160

**Published:** 2025-01-13

**Authors:** Fernando Ruiz Santiago, Lucía Bueno Caravaca, Francisco Garrido Sanz, Paula María Jiménez Gutiérrez, David Luengo Gómez, Mario Rivera Izquierdo, José Manuel Benítez, Antonio Jesús Láinez Ramos-Bossini

**Affiliations:** 1 Department of Musculoskeletal Radiology, Hospital Universitario Virgen de las Nieves, 18014 Granada, Spain; ferusan12@gmail.com (F.R.S.); luciabueno2@gmail.com (L.B.C.); davidluengog@gmail.com (D.L.G.); 2 Advanced Medical Imaging Group, Instituto de Investigación Biosanitaria de Granada (ibs.GRANADA), 18012 Granada, Spain; paulajg@correo.ugr.es (P.M.J.G.); j.m.benitez@decsai.ugr.es (J.M.B.); 3 Department of Radiology and Physical Medicine, University of Granada, 18016 Granada, Spain; 4 Department of Interventional Radiology, Hospital Universitario San Cecilio, 18007 Granada, Spain; fragarsan4@gmail.com; 5 Department of Anesthesiology, Hospital Universitario Virgen de las Nieves, 18014 Granada, Spain; 6 Department of Preventive Medicine and Public Health, School of Medicine, University of Granada, 18016 Granada, Spain; mariorivera@ugr.es; 7 Department of Computer Sciences and Artificial Intelligence, University of Granada, 18016 Granada, Spain; 8 Department of Human Anatomy and Embryology, University of Granada, 18016 Granada, Spain

**Keywords:** osteoporosis, fracture, spine, percutaneous vertebroplasty, conservative management, risk factor

## Abstract

**Objectives:** We aimed to analyze potential predictors for the development of metachronous fractures (MFs) after osteoporotic vertebral fractures (OVFs), with particular focus on radiological variables obtained at initial X-rays and computed tomography (CT) examinations, treatment applied (conservative management [CM] versus percutaneous vertebroplasty [PV]), and fractures located at the thoracolumbar junction (T11-L2). **Methods:** We conducted a two-center, observational retrospective study, including patients with single-level OVFs treated with CM or VP. We collected socio-demographic, radiological and treatment-related variables. We performed descriptive and contrastive bivariate analyses based on the presence of MFs and univariate and multivariate logistic regression analyses to obtain adjusted and crude odds ratios (aOR and cOR, respectively) for predicting MFs. Finally, we performed receiver-operating characteristic (ROC) curve analyses to determine the discriminative power of the models obtained. **Results:** Of the 90 patients included, 20 (22.2%) developed one or more MFs (15 in CM and 5 in PV groups, respectively; *p* = 0.037). The treatment group (aOR for PV, 0.087; 95%CI, 0.015–0.379), presence of intravertebral cleft (aOR, 5.62; 95%CI, 1.84–19.2) and difference in posterior height loss between X-rays and CT (aOR, 0.926; 95%CI, 0.856–0.992) were identified as significant predictors for MFs, while Genant’s numerical classification showed a trend toward significance (aOR, 1.97; 95%CI, 0.983–4.19; *p* = 0.064). A multivariate model combining these four variables showed optimal fitting and correctly discriminated over 80% of cases (AUC, 0.828; 95%CI, 0.725–0.930). Factors associated with MFs in thoracolumbar junction OVFs were intravertebral cleft, CM, posterior height loss in CT, and DGOU OF3 fractures. **Conclusions:** The presence of intravertebral cleft, a difference in posterior height loss between X-rays and CT equal to or lower than 2.4%, higher grades of Genant’s numerical classification, and application of CM instead of PV are predictors of MFs. These findings improve our understanding of the factors involved in the development of MFs, but they need to be validated prospectively.

## 1. Introduction

Osteoporotic vertebral fractures (OVFs) represent a health problem of high prevalence and increasing incidence due to the aging of the population [1,2]. These fractures may be associated with scarce or even absent symptoms, and some studies have suggested that only one-third of these fractures are clinically detected [3]. However, they are a major cause of morbidity, mortality and healthcare costs [1,4]. Up to 20% of OVFs may occur without previous trauma [2] and their presence increases the risk of metachronous vertebral fractures due to the highly deteriorated bone fragility in these patients [5,6].

There are different treatment options available for OVFs [7] and no universal agreement on their optimal management has been achieved to date. An increasing body of evidence emphasizes the need for adjusting the treatment strategy to the patient profile [8]. It is widely accepted that the first approach should be based on analgesic management and treating osteoporosis itself with hygienic–dietary and pharmacological measures [9,10]. However, there is no current consensus on the optimal therapeutic strategy for fractures. In most cases, conservative management (CM) is the first approach, although this decision may be contingent upon the fracture type, stability or presence of neurological deficits [11].

In general, CM consists of the use of rigid, semi-rigid and soft orthoses, which aim to minimize the shear forces on the fractured vertebrae. However, the scientific evidence supporting the usefulness of orthoses is limited [12]. In addition, CM is not adequately standardized, and up to one-third of patients with osteoporosis do not respond to it [13]. In cases refractory to CM, it is possible to perform vertebral augmentation techniques. Finally, more aggressive (i.e., surgical) techniques such as decompression of the central canal or vertebral fixation can be necessary, although the risks of the latter are considerable [9].

Percutaneous vertebroplasty (PV) was the first vertebral augmentation technique developed. It was performed in France in 1984 for the treatment of an aggressive hemangioma at C2 [14] and, by the end of the 1990s, it was widely adopted in clinical practice for the treatment of different types of vertebral fractures [15,16,17]. Briefly, PV is an imaging-guided percutaneous procedure by which a biological cement (usually polymethylmethacrylate) is injected into the fractured vertebral body, with the aim of relieving pain and increasing stability at the fracture site [9].

The efficacy of PV has been the subject of an ongoing debate since the publication of two randomized clinical trials in which no benefit was found in comparison with placebo in terms of pain control, improved functionality nor quality of life [18,19]. Likewise, some authors argue that PV may be responsible for the development of metachronous vertebral fractures (MFs) in adjacent vertebrae [20,21]. A relatively recent meta-analysis by Buchbinder et al. [22] concluded that there is no scientific evidence to recommend its use due to concerns related to both its efficacy and safety.

However, several clinical trials support the safety and efficacy of PV, as is the case with VAPOUR (2016), in which the time from fracture onset to PV was short [23], or the recent VERTOS V trial on chronic OVFs [24], which showed positive results favoring PV. Recent meta-analyses concluded that PV is effective, with proven benefits compared to CM, although the importance of using adequate selection criteria for patients has been stressed [25]. In this context, elderly people with recent, painful OVFs seem to be the most suitable patient profile that can benefit from PV [25,26].

Despite the increasing knowledge on the pathophysiology of OVFs, the question on whether PV increases the risk of MFs compared to CM remains unsolved. In addition, the identification of predictive factors involved in the development of metachronous OVFs is still limited. Some conditions (e.g., diabetes mellitus, chronic obstructive pulmonary disease), medications (e.g., corticosteroids), bone health indicators (e.g., bone mineral density) and demographic factors (e.g., age, sex, body mass index) have been linked to a higher risk of MFs [27,28]. However, radiological variables on X-rays and computed tomography (CT) images, which are the most frequently used imaging examinations when acute OVF is suspected due to their greater availability as compared to magnetic resonance imaging (MRI) [29,30], have been insufficiently explored in this setting. Of note, OVFs at the thoracolumbar junction deserve particular attention, as they have been reported to be a specific risk factor for higher kyphotic deformity and subsequent loss of sagittal balance, increasing the odds of developing MFs [31].

We previously reported that some radiological variables such as differences in posterior vertebral height loss between standing X-rays and CT or the fractured vs. non-fractured vertebral density ratio were associated with the development of vertebral collapse in OVFs treated with CM [32]. On the basis of these findings, we hypothesized that radiological variables from initial imaging examinations could be helpful for predicting the development of MFs and that the application of PV could be an important factor to be considered regarding this complication.

The main objective of this study was to identify potential predictors for the development of MFs after OVFs, with particular focus on radiological variables obtained at initial X-rays and CT examinations as well as the treatment applied (CM vs. PV). A secondary objective was to identify such predictors in the subgroup of patients with fractures located in the thoracolumbar junction.

## 2. Materials and Methods

### 2.1. Study Design and Patient Selection

A retrospective observational study was designed from a consecutive series of patients with acute OVF of the thoracolumbar spine diagnosed by imaging examinations at the Hospital Universitario Virgen de las Nieves and the Hospital Universitario San Cecilio between 1 January 2019 and 31 December 2023. The Strengthening the Reporting of Observational Studies in Epidemiology (STROBE) guidelines were followed in the design of this study [33]. This study was approved by the Provincial Ethics Committee of Granada (code TFG-FX-2019).

The following inclusion criteria were established:

1. Patients diagnosed with acute OVF by X-rays and CT.
2. Patients with radiological follow-up of their fracture at least 12 months after diagnosis.
3. Type A fractures of the AO Spine Classification [34,35].
4. Management with CM or PV (in the latter case, during the first 6 months after diagnosis).

The exclusion criteria were:

1. Lack of follow-up X-rays and/or CT at least 12 months after initial diagnosis.
2. Presence of two or more simultaneous OVFs.
3. Patients treated by spinal surgery prior to the diagnosis of OVF or during follow-up.
4. Patients with non-diagnostic-quality imaging examinations.

To ensure a balanced distribution of patients treated conservatively and with PV, patient selection was performed as follows: search filters were applied in the hospital radiology information system, which is common to both institutions involved, using the keywords “vertebral fracture” or “spinal fracture” (filter: all words). Single vertebral fracture cases were checked one by one and recorded in a spreadsheet. Next, the treatment applied was examined and classified according to whether it was fully conservative or with PV (in the first 6 months after diagnosis). Finally, the inclusion and exclusion criteria were applied consecutively by date of imaging examination and the first 45 patients treated conservatively, and the first 45 patients treated with PV were selected. Therefore, a total of 90 patients with OVF were analyzed in this study.

### 2.2. Study Variables

The dependent variable was the development of MFs on follow-up. The independent qualitative variables were sex, fracture location, cause (spontaneous, stress, fall), type of treatment applied (PV or CM), and presence of intravertebral cleft. In addition, we considered the type of fracture at initial diagnosis based on the following classification systems:

1. Morphological (wedge-shaped, biconcave, crush) and quantitative (grade 0, 1, 2, 3) classifications by Genant (34).
2. Classification of the German Society of Orthopedics and Traumatology (DGOU) (OF2-OF5) (35).
3. Classification of the AO Spine (A1 to A4) (32,33).
4. Sugita classification (swelled-front, bow-shaped, projecting, concave, dented) (36).

Independent quantitative variables were patient age, vertebral height loss in anterior, middle and posterior walls at diagnosis, difference in anterior, middle and posterior height loss between standing X-rays and supine CT images at diagnosis, density of the fractured and non-fractured vertebral bodies as well as fractured/non-fractured vertebrae and fractured vertebra/aorta density ratios.

**Figure 1:**
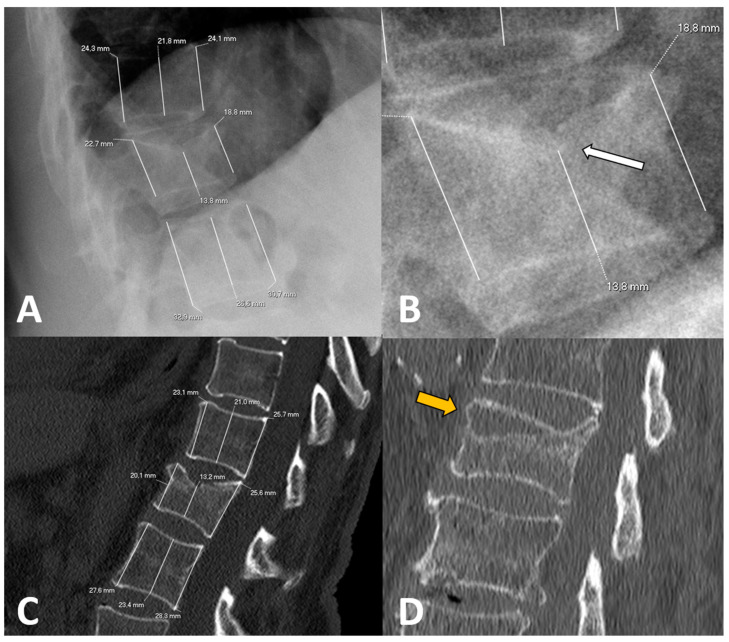
Examples of radiological variables assessed in our sample, from a 58-year-old woman (**A**–**C**) and an 88-year-old man (**D**). Patient 1. X-rays ((**A**), magnified in (**B**)) and CT at diagnosis (**C**). The height of each wall (anterior, middle and posterior) was measured through lines parallel to the anterior vertebral walls. Relative measurements were obtained for each fractured vertebral body by dividing them between the mean of the respective measurements in the cephalad and caudal vertebral bodies. Note that some cases involved subtle findings such as the asymmetric, focal endplate depression depicted with a white arrow in (**B**). Patient 2. CT at diagnosis (**D**) showing an obvious subchondral defect (orange arrow) consistent with a large intravertebral cleft.

To calculate vertebral height loss, the fractured vertebral body measurements were divided by the mean of the measurements made on the two adjacent vertebrae. To compare the variability between X-rays and CT measurements, the differences in height loss of each wall (i.e., anterior, middle and posterior) between both imaging techniques were calculated. Figure 1 shows illustrative examples of the measurements made.

**Figure 2:**
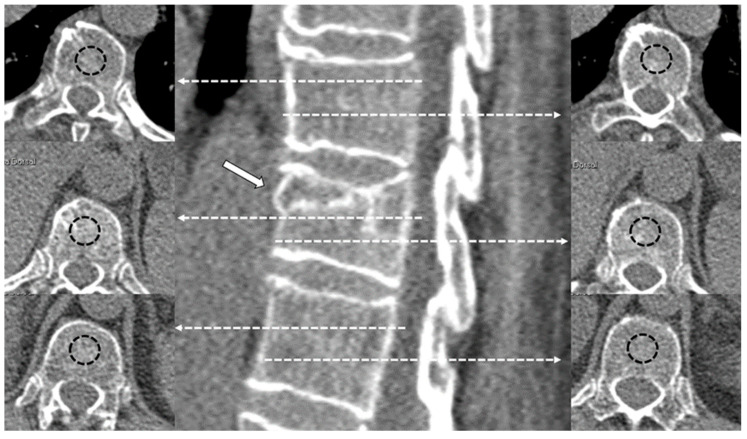
Examples of CT density measurements in a fractured vertebral body (arrow) and adjacent healthy vertebrae. The dashed lines with arrows indicate the level of different measurements in the axial plane. The dashed circles represent the region of interest (ROI) measured in the central part of the vertebral body.

As in our previous study [32], the values of CT density in Hounsfield Units (HU) [36] were measured by applying 1.5–2 cm^2^ oval region of interest (ROI) areas at two different levels of the trabecular bone of the fractured vertebra, in the two adjacent healthy vertebrae and in the aortic lumen. The mean value of these measurements was used as the final density value for the fractured vertebra, the normal vertebra and the aorta, respectively (Figure 2). For measurements in the fractured vertebra, cystic cavities or sclerotic lines of impaction were avoided. The aorta was chosen as the internal reference standard.

All CT scans were performed using 64-detector-row (Lightspeed VCT, General Electric^®^, Boston, MA, USA) and 128-detector-row (Ingenuity, Philips^®^, Amsterdam, The Netherlands) multidetector machines. CT images with a thickness of 0.63–1.25 mm and reconstructions with the same range were obtained. Two radiologists (ANONYMIZED and ANONYMIZED) with 1 and 7 years of experience, respectively, performed the measurements independently using the Carestream Vue system (Phillips^®^). The mean values of both measurements were used as final values. Fracture classification was also performed by both radiologists independently. In case of disagreement, the study was reviewed by a senior radiologist (ANONYMIZED) who decided the final classification for the fracture.

### 2.3. Statistical Analysis

A descriptive analysis was performed expressing qualitative variables as absolute and relative frequencies and quantitative variables as means and standard deviations. To analyze potential confounders that could be involved in the treatment applied, we performed both a univariate descriptive analysis for the whole sample and a bivariate descriptive analysis, including separated descriptive data for each of the treatment groups. This provided information on variables that could differ between patients managed with CM and PV.

Bivariate analyses were then performed to compare the group of patients who developed MFs and the group of patients who did not develop them. For these bivariate analyses, quantitative and qualitative variables were compared using Student’s *t*-tests and chi-squared tests (or Mann–Whitney/Fisher’s exact tests when the conditions to apply the respective parametric tests were not met).

Then, to analyze how the independent variables which showed statistically significant differences in the bivariate analyses comparing the categories of the treatment applied were able to predict the development of MFs, we performed univariate and multivariate binary logistic regression analyses. Crude and adjusted odds ratios (cOR and aOR, respectively) with their corresponding 95% confidence intervals (95%CIs) were obtained. For aORs related to the treatment applied, we adjusted the models for the variables that had resulted as significantly different in the corresponding bivariate descriptive analysis. To compare the consistency and fit of the multivariate models, we utilized the Akaike Information Criterion (AIC) and the Bayesian Information Criterion (BIC). Finally, receiver-operating characteristic (ROC) curves were used for discriminant analysis, and the Youden index was used to select the cut-off points that maximize the sensitivity and specificity of the variables of the models obtained.

As for the secondary objective of this study, all analyses were also performed for the subgroup of patients with fractures located at the thoracolumbar junction (T11-L2). All data were analyzed with the R software version 4.3.2 for Windows (Vienna, Austria). The level of statistical significance was established for *p* values less than 0.05.

## 3. Results

### 3.1. Main Characteristics of Patients and Vertebral Fractures in Our Sample

Of the 90 patients included in this study (mean age, 72.5 years; 74.4% women), the most frequent cause observed for OVFs was falling from standing height (80%), the level at which the OVF was most frequently located was L1 (37.8%), and the most frequent fracture types according to AO Spine, DGOU, Sugita and Genant’s morphological and Genant’s numerical classifications were, respectively, A1 (52.2%), OF2 (74.4%), bow-shaped (36.7%), biconcave (48.9%), and 0.5 and grade 1 (42.2%). Significant differences were observed in the distribution of categories in the AO Spine classification (*p* < 0.001), with a higher prevalence of Grade 0.5 fractures in the CT group compared to the PV group. In total, 20 patients (22.2%) developed one or more MFs on follow-up (15 in the conservatively treated group and 5 in the PV-treated group; *p* = 0.022). Table 1 shows the descriptive analysis of the socio-demographic variables, causes, location and type of the fractures included in this study. Supplementary Table S1 shows the same descriptive analysis for the subgroup of patients with OVFs located at the thoracolumbar junction.

Regarding the radiological variables measured, we observed baseline differences between the CT and PV groups in fractured vertebra density and fractured-to-healthy vertebral density ratio (higher in the PV group, *p* = 0.001 and *p* = 0.030, respectively), loss of posterior height measured in X-rays and CT (higher loss in the PV group in both, *p* = 0.009 and *p* = 0.031, respectively), and loss of anterior height measured in CT (higher loss in the PV group, *p* = 0.007). In addition, a higher loss of middle vertebral height was observed at the end of follow-up in the CT group (*p* = 0.019). The 12 other radiological variables showed no statistically significant differences between groups, although several trends were observed, indicating greater height loss in the PV group, in general. Table 2 shows the descriptive analysis of the radiological variables assessed in this study. Supplementary Table S2 shows the same descriptive analysis for the subgroup of patients with OVFs located at the thoracolumbar junction.

### 3.2. Variables Associated with the Development of Metachronous Vertebral Fractures

In the bivariate contrastive analyses comparing patients who developed MFs versus those who did not, in addition to the type of treatment applied (*p* = 0.022), we observed significant differences in the presence of intravertebral cleft (*p* = 0.004), difference in loss of posterior height between standing X-rays and CT (*p* = 0.042), and AO Spine fracture type classification (*p* = 0.028). Table 3 shows the results of the bivariate analyses. Supplementary Table S3 shows the same contrastive analyses for the subgroup of patients with OVFs located at the thoracolumbar junction.

### 3.3. Predictors for the Development of Metachronous Vertebral Fractures

The results of the univariate logistic regression analysis for MFs were statistically significant for the treatment group, intravertebral cleft, and difference in loss of posterior height between X-rays and CT. For the Genant’s classification (numerical), the results showed a trend toward significance (*p* = 0.064). In the multivariate logistic regression analyses, we analyzed two models, one with the three significant variables (“treatment”, “intravertebral cleft”, “difference in loss of posterior height between X-rays and CT”) and another one with the four variables (i.e., adding Genant’s classification). A comparison between the model with three variables and the model with four variables showed that AIC decreased from 87.26 to 81.74 and BIC decreased from 97.26 to 94.24, indicating that the inclusion of the latter variable improved model fit, with an optimal balance between quality of fit and model complexity. Table 4 shows the results of the univariate and multivariate regression analyses. Supplementary Table S4 shows the same contrastive analyses for the subgroup of patients with OVFs located at the thoracolumbar junction.

### 3.4. Discriminative Power of the Models for Predicting Metachronous Fractures

**Figure 3:**
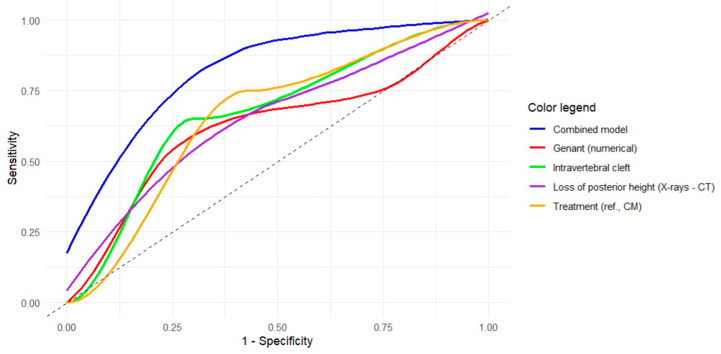
Receiver operating characteristic (ROC) curves of the univariate and multivariate models obtained for the prediction of metachronous vertebral fractures. The colored curves represent the ROC curves for each significant independent variable based on the univariate logistic regression results (see legend). The blue curve refers to the multivariate model with 4 variables. The gray diagonal line corresponds to the reference of a random classification (line of no discrimination).

The results of the ROC curve analyses for each of the significant models are shown in Table 5 and represented in Figure 3. For the variable “loss of posterior height (X-rays–CT)”, the optimal cutoff of 0.240 (Youden index = 0.328) showed a sensitivity of 60% and a specificity of 72.9% to predict MFs. Supplementary Table S5 and Supplementary Figure S1 show the same analyses and graphical representation for the subgroup of patients with OVFs located at the thoracolumbar junction. For the variable “loss of posterior height (CT)”, the optimal cutoff of 0.161 (Youden index = 0.447) showed a high sensitivity (92.9%) but a low specificity (51.9%) to predict MFs.

**Figure 4:**
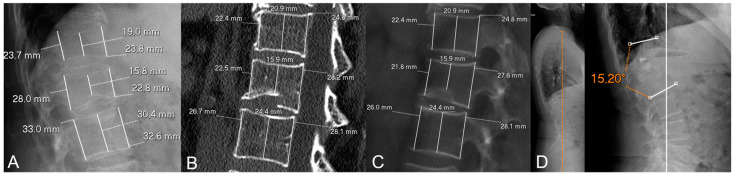
Illustrative example of a patient without metachronous fractures in our sample. (**A**) Initial X-rays. (**B**) Initial CT (MPR set at 1 mm). (**C**) Initial CT (MPR set at 100 mm for easier comparison with measurements on X-rays). (**D**) Follow-up X-rays. This is a 77-year-old woman with a biconcave, grade 1 (Genant’s classification), OF2 (DGOU’s classification) fracture of L1. The loss of posterior vertebral height (PVH) on CT was negative and the difference in loss of PVH was close to 0 (0.1%). She was treated with vertebroplasty (note a slight cement leak at T12-L1 intervertebral disc) and developed no metachronous fractures. In addition, follow-up X-rays 3 years later showed low-to-moderate local kyphosis (monosegmental Cobb angle of 15°) with preservation of sagittal balance (orange vertical line in (**D**)).

**Figure 5:**
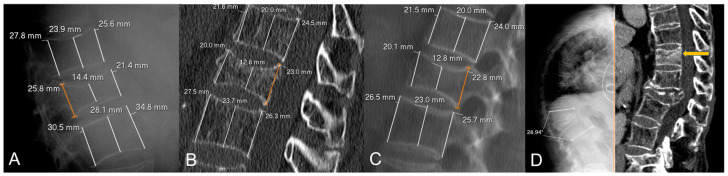
Illustrative example of a patient with metachronous fracture in our sample. (**A**) Initial X-rays. (**B**) Initial CT (MPR set at 1 mm). (**C**) Initial CT (MPR set at 100 mm for easier comparison with measurements on X-rays). (**D**) Follow-up X-rays. This is a 74-year-old woman with a biconcave, grade 2 (Genant’s classification), OF2 (DGOU’s classification) involving L1. The loss of posterior vertebral height (PVH) on CT was 9.5% and the difference in loss of PVH between X-rays and CT was 2%. She was managed conservatively and developed vertebral collapse and hyperkyphosis (monosegmental Cobb angle close to 30°) with loss of sagittal balance (sagittal vertical axis shown as an orange vertical line at the left panel in (**D**)). At 2-year follow-up, she developed L1 collapse and a metachronous fracture of T11 (yellow arrow in (**D**)).

Figure 4 and Figure 5 show illustrative examples of patients from our sample with and without MFs following OVFs at the thoracolumbar junction. Other illustrative examples in patients with OVFs in locations different from the thoracolumbar junction are provided in Supplementary Figures S2 and S3.

## 4. Discussion

This study aimed to identify factors associated with the development of new incident OVFs (i.e., MFs), with particular focus on a comparison between the differential effects of PV and CM. As a secondary objective, we analyzed factors associated with MFs in patients with fractures located at the thoracolumbar junction. In the whole sample, we found that the presence of an intravertebral cleft and a difference in posterior height loss between X-rays and CT in initial imaging examinations were significantly associated with a higher risk of developing MFs, and a trend toward significance for the type of fracture according to Genant’s numerical classification was observed. Conversely, PV was associated with a significantly lower risk of subsequent OVFs, indicating a protective effect. A combined model including four variables showed a moderate-to-high discriminant value for predicting the development or not of MFs. In fractures located at the thoracolumbar junction, we classified the fracture type according to DGOU; a higher loss of posterior height at CT and the presence of intravertebral cleft increased the risk of developing MFs, while PV showed a protective effect. A multivariate model combining three of these variables also showed a moderate-to-high discriminant value for predicting the development or not of MFs. However, there are some limitations that need to be considered, particularly derived from the retrospective nature of this study and the relatively limited sample size, which probably underpowered the statistical analyses.

Regarding radiological measurements, first of all, we found that the presence of intravertebral cleft increased the chance of developing MFs, with higher OR values in fractures located at the thoracolumbar junction. This is consistent with previous findings. For instance, a study by Trout et al. found that patients treated with PV for fractures with clefts had a nearly twofold (OR, 1.90; 95%CI, 1.04–3.49) increased risk of subsequent fractures compared to those without clefts [37]. Our results showed an even more pronounced association of over a fourfold increase in such a risk. Similarly, Wang et al. identified intravertebral clefts as a significant risk factor for adjacent-level symptomatic fractures after vertebral augmentation, with a higher incidence of new fractures within six months post-procedure in patients with clefts [38]. However, to our knowledge, no specific studies have assessed the risk of MFs in OVFs with intravertebral clefts managed conservatively, although in these patients, clefts are a known risk factor for increasing vertebral collapse [31]. Considering that PV was found to have a significant protective effect for MFs in our sample, further studies in patients managed conservatively are warranted to determine how the presence of clefts in acute OVFs influences the prognosis and risk of MFs in these patients.

Secondly, we observed that involvement of the posterior wall increases the risk of developing MFs. In the whole sample, the loss of posterior vertebral height between standing X-rays and CT showed an inverse relationship with the risk of MFs, with an optimal cutoff point of 2.4%. Interestingly, this pattern is opposite to the one found in our previous study for predicting vertebral collapse [32]. Although the significance of this finding from a pathophysiological perspective is unclear, we hypothesize that increased tendency of the posterior wall to collapse could favor a more homogeneous distribution of load forces, decreasing kyphotic angulation in the mid or long term, which is a known risk factor for the development of MFs. In fact, Okamoto et al. demonstrated through finite element analysis that kyphotic deformity significantly increases compressive stresses on adjacent vertebrae, thereby elevating the risk of subsequent fractures [39]. Similarly, Huang et al. found that hyperkyphotic posture, which can result from increased local kyphosis, is associated with a higher risk of MFs [40]. The association between vertebral collapse and MFs considering kyphotic changes in the light of these findings should also be explored in future studies. Notably, this trend was also observed in the subgroup of patients with fractures located at the thoracolumbar junction, but it did not reach statistical significance, probably owing to the more reduced sample size. Conversely, for these patients, our results showed that a higher loss of posterior vertebral height at initial CT was a predictor for developing MFs. Overall, these findings highlight the relevance of posterior wall involvement in spinal stability and call for more profound analyses in future studies.

On the other hand, we observed an association between the categories of Genant’s numerical classification and MFs in the whole sample, with a higher likelihood of developing MFs as the degrees of this classification increase. However, it should be taken into account that there was an asymmetric distribution in the fracture categories between the treatment groups, with a significantly higher number of OVFs grade 0.5 in the CM group. Therefore, this finding may represent a confounding effect, explainable by the fact that OVFs with minimal height loss are more prone to be treated conservatively rather than with PV (at least in the short or mid term). This is supported by the results of our multivariate analysis, where the inclusion of the Genant’s classification, which showed a trend toward significance in the univariate regression analysis, improved model fitting. The other OVF classifications analyzed showed no significant associations with the risk of developing MFs, in line with other previous studies [41,42]. Interestingly, although we observed a similar trend for the Genant’s numerical classification in the subgroup of patients with OVFs at the thoracolumbar junction, it did not reach statistical significance (*p* = 0.058). Conversely, the DGOU classification, which showed a trend toward significance in the whole sample, did, probably due to the fact that only OF2 and OF3 fractures (i.e., no OF4 fractures) were present in patients with OVFs at the thoracolumbar junction. A higher risk of developing MFs was found in OF3 fractures compared to OF2 fractures, emphasizing the role of posterior wall involvement, in line with our previously described findings. These findings underscore the need to find classification systems with greater prognostic capacity for the development of OVFs.

Finally, probably the most interesting result that we observed is that PV was associated with a significant decrease in the risk of developing metachronous OVFs compared to CM. Specifically, the incidence of new incident OVFs in patients treated with PV occurred in 5 cases (11.1%), while in the CM group, it occurred in 15 cases (33.3%), with statistically significant differences and a protective cOR of 0.250 and aOR of 0.050. These outcomes were analogous in the subgroup of patients with fractures located at the thoracolumbar junction, with 3 (9.7%) and 11 (29.7) MFs in patients treated with PV and CM, respectively (*p* = 0.042), and cOR and aOR values of 0.253 and 0.120, respectively. Of note, for aOR, we adjusted for variables that showed significant differences between patients managed with CM and PV (potential confounders), apart from sex and age. The main biological rationale for this finding lies in the decreased destabilization of the initial fracture focus and corroborates the results of previous studies [43,44] while disagreeing with authors who argued that the risk of developing MFs in adjacent vertebrae increases due to the stiffness and biomechanical change caused by PV [45,46]. As in previous meta-analyses [44,47], our findings defy the hypothesis that PV contributes to increased risk of subsequent vertebral fracture but emphasize the need for adequately designed randomized controlled trials to confirm these findings.

The main limitations of this study lie in its retrospective nature, in not having assessed some potential confounders such as bone mineral density, and in the relatively limited sample size, which probably underpowered some statistical analyses and precluded level-by-level contrastive analyses, particularly in the subgroup of patients with fractures located at the thoracolumbar junction. Notably, the discrepancies observed in the predictivity of some radiological variables in the whole sample as compared to patients with fractures in the thoracolumbar junction, which probably owed to variations in both the sample sizes and the specific features of vertebrae in different spinal segments, merit further investigation. The main advantages of this study lie in the high number of radiological variables measured by expert musculoskeletal radiologists, its two-centered nature, and the long follow-up (mean follow-up over 2 years). Overall, it is necessary to validate our results in prospective studies with larger sample sizes.

## 5. Conclusions

In osteoporotic vertebral fractures of the thoracic and lumbar spine, the presence of an intravertebral cleft, a difference in loss of posterior vertebral height between standing X-rays and CT equal to or lower than 2.4%, and the application of conservative treatment instead of vertebroplasty were identified as potential predictors for the development of metachronous vertebral fractures. In fractures located at the thoracolumbar junction, the most relevant predictors were intravertebral cleft, posterior height loss compared to adjacent vertebrae at initial CT over 1.6%, OF3 fractures of the DGOU’s classification, and conservative management. These findings could improve our understanding of the course of osteoporotic vertebral fractures and improve patient selection for treatment-based strategies. However, some limitations of our study, particularly derived from its retrospective nature, call for prospective validation in future studies with larger sample sizes.

## Figures and Tables

**Table 1:** Descriptive analysis of the socio-demographic variables, causes, location and types of the fractures. Data are expressed as mean ± standard deviation or absolute (relative) frequencies for quantitative and qualitative variables, respectively. * Significant *p*-value.

Variable	Total Sample (*N* = 90) X ± SD/*N* (%)	Vertebroplasty (*n* = 45) X ± SD/*N* (%)	Cons. Management (*n* = 45) X ± SD/*N* (%)	*p*-Value
Sex (female)	67 (74.4)	31 (68.9)	36 (80)	0.227
Age	72.5 ± 9.5	72.6 ± 10.3	72.3 ± 8.8	0.860
Follow-up	24.6 ± 20.9	27.3 ± 26.3	21.9 ± 13.3	0.226
Cause of Fracture				0.629
Fall from standing height	77 (85.6)	37 (82.2)	40 (88.9)	
Spontaneous	6 (6.7)	4 (8.9)	2 (4.4)	
Stress	7 (7.8)	4 (8.9)	3 (6.7)	
Fracture Level				0.315
T7	3 (3.3)	2 (4.4)	1 (2.2)	
T8	3 (3.3)	3 (6.7)	0 (0)	
T11	8 (8.9)	4 (8.9)	4 (8.9)	
T12	13 (14.4)	7 (15.6)	6 (13.3)	
L1	34 (37.8)	12 (26.7)	22 (48.9)	
L2	13 (14.4)	8 (17.8)	5 (11.1)	
L3	7 (7.8)	4 (8.9)	3 (6.7)	
L4	7 (7.8)	3 (6.7)	4 (8.9)	
L5	2 (2.2)	2 (4.4)	0 (0)	
Fracture Type AO Spine				0.446
A1	47 (52.2)	23 (51.1)	24 (53.3)	
A2	2 (2.2)	2 (4.4)	0 (0)	
A3	29 (32.2)	13 (28.9)	16 (35.6)	
A4	12 (13.3)	7 (15.6)	5 (11.1)	
Fracture Type DGOU				0.602
OF2	67 (74.4)	33 (73.3)	34 (75.6)	
OF3	22 (24.4)	11 (24.4)	11 (24.4)	
OF4	1 (1.1)	1 (2.2)	0 (0)	
Fracture Type Sugita				0.381
Swelled-front	19 (21.1)	13 (28.9)	6 (13.3)	
Bow-shaped	33 (36.7)	16 (35.6)	17 (37.8)	
Projecting	12 (13.3)	4 (8.9)	8 (17.8)	
Concave	21 (23.3)	10 (22.2)	11 (24.4)	
Dented	5 (5.6)	2 (4.4)	3 (6.7)	
Fracture Type Genant m.				0.580
Wedge	42 (46.7)	21 (46.7)	23 (51.1)	
Biconcave	44 (48.9)	21 (46.7)	21 (46.7)	
Crush	4 (4.4)	3 (6.7)	1 (2.2)	
Fracture Type Genant n.				<0.001 *
0.5	23 (25.6)	1 (2.2)	22 (48.9)	
1	38 (42.2)	29 (64.4)	9 (20)	
2	29 (32.2)	15 (33.3)	14 (31.1)	
Metachronous fractures	20 (22.2)	5 (11.1)	15 (33.3)	0.022 *

**Table 2:** Descriptive analysis of the radiological variables assessed in the study with bivariate descriptive analysis based on the treatment that was applied. Data are expressed as mean ± standard deviation or absolute (relative) frequencies for quantitative and qualitative variables, respectively. * Significant *p*-value.

Variable	Total Sample (*N* = 90) X ± SD/*N* (%)	Vertebroplasty (*n* = 45) X ± SD/*N* (%)	Cons. Management (*n* = 45) X ± SD/*N* (%)	*p*-Value
Intravertebral cleft	34 (37.8)	13 (28.9)	21 (46.7)	0.082
Healthy vertebra density	87.3 ± 32.3	88.6 ± 33.0	86.0 ± 32.0	0.703
Fractured vertebra density	139.6 ± 52.0	158.4 ± 54.1	120.8 ± 42.7	<0.001 *
Aorta density	42.3 ± 14.5	42.1 ± 8.2	39.8 ± 5.0	0.124
Fracture/non-fracture density ratio	1.9 ± 1.5	2.1 ± 1.9	1.6 ± 0.7	0.062
Fracture/aorta density ratio	3.5 ± 1.5	3.8 ± 1.5	3.1 ± 1.4	0.030
Loss of anterior height (X-rays)	26.1 ± 15.8	29.3 ± 17.8	22.9 ± 12.9	0.054
Loss of middle height (X-rays)	29.9 ± 12.3	32.4 ± 13.2	27.4 ± 11.0	0.052
Loss of posterior height (X-rays)	9.5 ± 10.3	12.3 ± 11.1	6.7 ± 8.7	0.009 *
Loss of anterior height (CT)	19.5 ± 14.1	23.5 ± 14.9	15.6 ± 12.1	0.007 *
Loss of middle height (CT)	25.5 ± 14.1	28.1 ± 15.5	22.8 ± 12.2	0.074
Loss of posterior height (CT)	7.8 ± 8.5	9.7 ± 10.0	5.9 ± 6.1	0.032 *
Loss of anterior height (X-rays–CT)	6.6 ± 9.2	5.9 ± 8.9	7.4 ± 9.7	0.436
Loss of middle height (X-rays–CT)	4.4 ± 11.5	4.3 ± 11.2	4.6 ± 12.0	0.912
Loss of posterior height (X-rays–CT)	1.7 ± 8.2	2.6 ± 7.3	0.8 ± 8.9	0.305

**Table 3:** Factors associated with the development of new osteoporotic vertebral fractures. Data are expressed as mean ± standard deviation or absolute (relative) frequencies. * Significant *p*-value.

Variable	Total Sample (*N* = 90)	New Fractures (*n* = 20)	No New Fractures (*n* = 70)	*p*-Value
Treatment				0.022 *
Percutaneous vertebroplasty	45 (50)	5 (25)	40 (57.1)	
Conservative management	45 (50)	15 (75)	30 (42.9)	
Sex (Female)	67 (74.4)	17 (85)	50 (71.4)	0.220
Age	72.5 ± 9.5	71.6 ± 7.3	72.7 ± 10.1	0.564
Follow-up	24.6 ± 20.9	23.5 ± 15.0	24.9 ± 22.4	0.784
Cause				0.380
Spontaneous	6 (6.7)	0 (0)	6 (8.6)	
Stress	7 (7.8)	2 (10)	5 (7.1)	
Fall from standing height	77 (85.5)	18 (90)	59 (84.3)	
Fracture Type (AO Spine)				0.162
A1	47 (52.2)	7 (35)	40 (57.1)	
A2	2 (2.2)	0 (0)	2 (2.9)	
A3	29 (32.2)	8 (40)	21 (30)	
A4	12 (13.3)	5 (25)	7 (7.8)	
Fracture Type (DGOU)				0.057
OF2	67 (74.4)	11 (55)	56 (80)	
OF3	22 (24.4)	9 (45)	13 (18.6)	
OF4	1 (1.1)	0 (0)	1 (1.4)	
Fracture Type (Sugita)				0.501
Swelled-front	19 (21.1)	3 (15)	16 (22.9)	
Bow-shaped	33 (36.7)	9 (45)	24 (34.3)	
Projecting	12 (13.3)	4 (20)	8 (11.4)	
Concave	21 (23.3)	4 (20)	17 (24.3)	
Dented	5 (5.6)	0 (0)	5 (7.1)	
Fracture Type (Genant morphol.)				0.665
Wedge	42 (46.7)	11 (55)	36 (51.4)	
Biconcave	44 (48.9)	8 (40)	31 (44.3)	
Crush	4 (4.4)	1 (5)	3 (4.3)	
Fracture type (Genant numerical)				0.028 *
0.5	23 (25.6)	5 (25)	18 (25.7)	
1	38 (42.2)	4 (20)	34 (48.6)	
2	29 (32.2)	11 (55)	13 (18.6)	
Intravertebral cleft	34 (37.8)	13 (65)	21 (30)	0.004 *
Healthy vertebra density	87.3 ± 32.3	89.6 ± 30.5	79.2 ± 37.8	0.209
Fractured vertebra density	139.6 ± 52.0	136.9 ± 52.1	149.1 ± 51.9	0.360
Aorta density	42.3 ± 14.5	40.6 ± 5.6	42.2 ± 10.2	0.378
Fracture-to-healthy vertebra density ratio	1.9 ± 1.5	1.8 ± 1.5	2.2 ± 1.2	0.205
Fracture-to-aorta density ratio	3.5 ± 1.5	3.4 ± 1.4	3.5 ± 1.8	0.067
Loss of anterior height (X-rays)	26.1 ± 15.8	24.9 ± 16.5	30.5 ± 12.2	0.162
Loss of middle height (X-rays)	29.9 ± 12.3	30.2 ± 12.4	28.9 ± 12.4	0.677
Loss of posterior height (X-rays)	9.5 ± 10.3	9.4 ± 10.4	9.5 ± 10.4	0.961
Loss of anterior height (CT)	19.5 ± 14.1	18.2 ± 14.8	24.2 ± 10.2	0.093
Loss of middle height (CT)	25.5 ± 14.1	24.7 ± 14.5	28.2 ± 12.7	0.329
Loss of posterior height (CT)	7.8 ± 8.5	10.9 ± 7.6	6.9 ± 8.5	0.058
Loss of anterior height (X-rays–CT)	6.6 ± 9.2	6.7 ± 9.3	6.3 ± 9.4	0.873
Loss of middle height (X-rays–CT)	4.4 ± 11.5	5.5 ± 11.2	0.7 ± 12.1	0.100
Loss of posterior height (X-rays–CT)	1.7 ± 8.2	−1.5 ± 8.0	2.6 ± 8.0	0.042 *

**Table 4:** Univariate and multivariate logistic regression analyses for the prediction of metachronous fractures including the variables that showed statistically significant differences in the bivariate analyses. CM, Conservative management. OR, odds ratio. cOR, crude OR. aOR: adjusted OR. 95%CI, 95% confidence interval. ^U^ *p*-value of the univariate regression analysis. ^M^ *p*-value of the multivariate regression analysis. * Significant *p*-value. ^ For this variable, apart from sex and age, adjustment for the six variables that showed statistically significant differences in the bivariate descriptive analyses of Table 1 and Table 2 was made.

Variable	cOR [95%CI]	*p*-Value ^U^	aOR [95%CI]	*p*-Value ^M^
Treatment [Ref: CM]	0.250 [0.075–0.724]	0.015	0.050 [0.006–0.253] ^	0.001 *
Genant (numerical)	1.720 [0.879–3.560]	0.123	1.97 [0.983–4.190]	0.064
Intravertebral cleft	4.330 [1.550–13.000]	0.006	5.62 [1.840–19.200]	0.003 *
Loss of posterior height (X-rays–CT)	0.930 [0.862–0.995]	0.047	0.926 [0.856–0.992]	0.038 *

**Table 5:** Receiver-operating characteristic curve analysis for the variables of the multivariate model to predict the development of new fractures. The combined model with 3 variables includes the variables “treatment”, “intravertebral cleft”, “difference in loss of posterior height between X-rays and CT”. The combined model with 4 variables includes these variables as well as “Genant’s classification (numerical)”. AUC, area under the curve. 95%CI, 95% confidence interval. * Significant *p*-value.

Variable	AUC [95%CI]	*p*-Value
Treatment [Ref: CM]	0.660 [0.547–0.774]	0.012 *
Genant (numerical)	0.611 [0.462–0.761]	0.107
Intravertebral cleft	0.675 [0.555–0.795]	0.005 *
Loss of posterior height (X-rays–CT)	0.660 [0.519–0.802]	0.030 *
Combined model (3 variables)	0.781 [0.672–0.891]	<0.001 *
Combined model (4 variables)	0.828 [0.725–0.930]	<0.001 *

## Data Availability

The data presented in this study are available on request from the corresponding author due to restrictions imposed by the Ethics Committee, which approved the study protocol.

## Supplementary Materials

The following supporting information can be downloaded at: https://www.mdpi.com/article/10.3390/diagnostics15020160/s1, Supplementary Table S1. Descriptive analysis of the socio-demographic variables, causes, location and types of the fractures located at the thoracolumbar junction; Supplementary Table S2. Descriptive analysis of the radiological variables assessed in the study for fractures located at the thoracolumbar junction with bivariate descriptive analysis based on the treatment that was applied; Supplementary Table S3. Factors associated with the development of new osteoporotic vertebral fractures in patients with fractures located at the thoracolumbar junction; Supplementary Table S4. Univariate and multivariate logistic regression analyses for the prediction of metachronous fractures in patients with vertebral fractures located at the thoracolumbar junction, including the variables that showed statistically significant differences in the bivariate analyses; Supplementary Table S5. Receiver-operating characteristics curve analysis for the variables of the multivariate model to predict the development of new fractures. The combined model includes the variables “treatment”, DGOU classification and loss of posterior height; Supplementary Figure S1. ROC curves of the univariate and multivariate models obtained for the prediction of metachronous vertebral fractures in patients with fractures located at the thoracolumbar junction; Supplementary Figure S2. Illustrative example of a patient without metachronous fractures in our sample; Supplementary Figure S3. Illustrative example of a patient who developed a metachronous fracture in our sample.
