# Supplementary material for: Factors Influencing the Development of Metachronous Fractures in Patients with Osteoporotic Vertebral Fractures Treated with Conservative Management or Vertebroplasty
Source: Diagnostics (Basel). 2025 Jan 13;15(2):160. doi: 10.3390/diagnostics15020160 (PMC11765220; doi:10.3390/diagnostics15020160)

diagnostics

MDPI

Supplementary Files

Supplementary Table S1. Descriptive analysis of the socio-demographic variables, causes, location and types of the fractures located at the thoracolumbar junction. Data are expressed as mean + standard deviation or absolute (relative) frequencies for quantitative and qualitative variables, respectively. * Significant p-value.

Variable	Total sample (N = 68) X±SD / N (%)	Vertebroplasty (n = 31) X±SD / N (%)	Cons. management (n = 37) X±SD / N (%)	P-value
Sex (female)	51 (75.0)	23 (74.2)	28 (75.7)	0.888
Age	72.4 ± 9.8	72.7 ± 10.6	72.1 ± 9.2	0.802
Follow-up	21.8 ± 14.6	21.1 ± 16.7	22.3 ± 12.7	0.745
**Cause of Fracture**				
Fall from standing height	60 (88.2)	27 (87)	33 (89.2)	0.965
Spontaneous	4 (5.9)	2 (6.5)	2 (5.4)	
Stress	4 (5.9)	2 (6.5)	2 (5.4)	
**Fracture Level**				
T11	8 (11.8)	4 (12.9)	4 (10.8)	0.361
T12	13 (19.1)	7 (22.6)	6 (16.2)	
L1	34 (50.0)	12 (38.7)	22 (59.5)	
L2	13 (19.1)	8 (25.8)	5 (13.5)	
Fracture Type AO Spine				
A1	39 (57.4)	18 (58.1)	21 (56.8)	0.562
A2	1 (1.5)	1 (3.2)	0 (0)	
A3	21 (30.9)	8 (25.8)	13 (35.1)	
A4	7 (10.3)	4 (12.9)	3 (8.1)	
Fracture Type DGOU				
OF2	52 (76.5)	25 (80.6)	27 (73)	0.458
OF3	16 (23.5)	6 (19.4)	10 (27)	
Fracture Type Sugita				
Swelled-front	14 (20.6)	10 (32.3)	4 (10.8)	0.273
Bow-shaped	24 (35.3)	9 (29)	15 (40.5)	
Projecting	9 (13.2)	3 (9.7)	6 (16.2)	
Concave	18 (26.5)	8 (25.8)	10 (27)	
Dented	3 (4.4)	1 (3.2)	2 (5.4)	
Fracture Type Genant m.				0.517
Wedge	35 (51.5)	15 (48.4)	20 (54.1)	
Biconcave	32 (47.1)	15 (48.4)	20 (54.1)	
Crush	1 (1.5)	1 (3.2)	0 (0)	
Fracture Type Genant n.				
0.5	15 (22.1)	0 (0)	15 (40.5)	**<0.001***
1	32 (47.1)	23 (74.2)	9 (24.3)	
2	21 (30.9)	8 (25.8)	13 (35.1)	
Metachronous fractures	14 (20.6)	3 (9.7)	11 (29.7)	0.042*

Supplementary Table S2. Descriptive analysis of the radiological variables assessed in the study for fractures located at the thoracolumbar junction with bivariate descriptive analysis based on the treatment that was applied. Data are expressed as mean + standard deviation or absolute (relative) frequencies for quantitative and qualitative variables, respectively. * Significant p-value.

**Variable**	**Total sample (N = 68) X±SD / N (%)**	**Vertebroplasty (n = 31) X±SD / N (%)**	**Cons. management (n = 37) X±SD / N (%)**	**P-value**
Intravertebral cleft	28 (41.2)	11 (35.5)	17 (45.9)	0.383
Healthy vertebra density	91.2 ± 31.6	91.0 ± 32.7	91.3 ± 31.2	0.975
Fractured vertebra density	136 ± 44.1	150.9 ± 43.3	123.5 ± 41.3	**0.009***
Aorta density	40.2 ± 5.2	40.8 ± 5.0	39.6 ± 5.4	0.363
Fracture/non-fracture density ratio	1.6 ± 0.8	1.8 ± 0.9	1.5 ± 0.7	0.070
Fracture/aorta density ratio	3.4 ± 1.3	3.6 ± 1.3	3.2 ± 1.4	0.184
Loss of anterior height (X-rays)	26.3 ± 14.7	28.6 ± 16.4	24.4 ± 12.9	0.254
Loss of middle height (X-rays)	29.5 ± 12.5	31.3 ± 13.3	28.0 ± 11.7	0.287
Loss of posterior height (X-rays)	9.3 ± 10.1	11.4 ± 10.8	7.5 ± 9.3	0.114
Loss of anterior height (CT)	19.0 ± 11.5	21.9 ± 12.1	16.6 ± 10.5	0.061
Loss of middle height (CT)	24.4 ± 13.2	25.1 ± 14.9	23.8 ± 11.8	0.688
Loss of posterior height (CT)	7.4 ± 7.3	8.7 ± 8.6	6.4 ± 6.0	0.229
Loss of anterior height (X-rays – CT)	7.3 ± 9.6	6.7 ± 9.2	7.8 ± 10.0	0.633
Loss of middle height (X-rays – CT)	5.2 ± 11.8	6.2 ± 11.5	4.3 ± 12.2	0.496
Loss of posterior height (X-rays – CT)	1.9 ± 8.6	2.8 ± 7.7	1.1 ± 9.4	0.403

Supplementary Table S3. Factors associated with the development of new osteoporotic vertebral fractures in patients with fractures located at the thoracolumbar junction. Data are expressed as mean ± standard deviation or absolute (relative) frequencies. *Significant p-value.

Variable	Total sample (N = 68)	New fractures (n = 14)	No new fractures (n = 54)	P-value
**Treatment**				
Percutaneous vertebroplasty	31 (45.6)	3 (21.4)	28 (51.9)	**0.042***
Conservative management	37 (54.4)	11 (78.6)	26 (48.1)	
Sex (Female)	51 (75.0)	11 (78.6)	40 (74.1)	0.729
Age	72.4 ± 9.8	72.6 ± 8.1	72.3 ± 10.2	0.912
Follow-up	21.8 ± 14.6	20.3 ± 13.2	22.1 ± 15.0	0.673
**Cause**				
Spontaneous	60 (88.2)	0 (0)	4 (7.4)	
Stress	4 (5.9)	1 (7.1)	3 (5.6)	
Fall from standing height	4 (5.9)	13 (92.9)	47 (87.0)	0.569
**Fracture Type (AO Spine)**				
A1	39 (57.4)	5 (35.7)	34 (63.0)	
A2	1 (1.5)	0 (0)	1 (1.9)	
A3	21 (30.9)	6 (42.9)	15 (27.8)	
A4	7 (10.3)	3 (21.4)	4 (7.4)	0.200
**Fracture Type (DGOU)**				
OF2	52 (76.5)	6 (42.9)	46 (85.2)	
OF3	16 (23.5)	8 (57.1)	8 (14.8)	**0.001***
**Fracture Type (Sugita)**				
Swelled-front	14 (20.6)	2 (14.3)	12 (22.2)	
Bow-shaped	24 (35.3)	7 (50)	17 (31.5)	
Projecting	9 (13.2)	2 (14.3)	7 (13.0)	
Concave	18 (26.5)	3 (21.4)	15 (27.8)	
Dented	3 (4.4)	0 (0)	3 (5.6)	0.666
**Fracture Type (Genant morph.)**				
Wedge	35 (51.5)	9 (64.3)	26 (58.1)	
Biconcave	32 (47.1)	5 (35.7)	27 (50.0)	
Crush	1 (1.5)	0 (0)	1 (1.9)	0.521
**Fracture type (Genant num.)**				
0.5	15 (22.1)	2 (14.3)	13 (24.1)	
1	32 (47.1)	4 (28.6)	28 (51.9)	
2	21 (30.9)	8 (57.1)	13 (24.1)	0.058
Intravertebral cleft	28 (41.2)	10 (71.4)	18 (33.3)	**0.010***
Healthy vertebra density	91.2 ± 31.6	93.8 ± 28.7	81.2 ± 40.6	0.186
Fractured vertebra density	136 ± 44.1	145.1 ± 50.3	133.6 ± 42.6	0.391
Aorta density	40.2 ± 5.2	39.3 ± 4.6	40.4 ± 5.3	0.475
Fracture/non-fracture density ratio	1.6 ± 1.3	2.2 ± 1.3	1.5 ± 0.5	0.081
Fracture/aorta density ratio	3.4 ± 1.3	3.6 ± 2.0	3.4 ± 1.1	0.569
Loss of anterior height (X-rays)	26.3 ± 14.7	31.1 ± 13.1	25.0 ± 14.9	0.168
Loss of middle height (X-rays)	29.5 ± 12.5	29.0 ± 14.3	29.7 ± 12.1	0.855
Loss of posterior height (X-rays)	9.3 ± 10.1	9.9 ± 11.0	9.1 ± 10.0	0.828
Loss of anterior height (CT)	19.0 ± 11.5	23.4 ± 11.1	17.8 ± 11.4	0.108
Loss of middle height (CT)	24.4 ± 13.2	28.0 ± 14.1	23.4 ± 12.9	0.247
Loss of posterior height (CT)	7.4 ± 7.3	11.4 ± 5.9	6.4 ± 7.3	**0.013***
Loss of anterior height (X-rays – CT)	7.3 ± 9.6	7.7 ± 9.8	7.2 ± 9.6	0.851
Loss of middle height (X-rays – CT)	5.2 ± 11.8	1.0 ± 13.2	6.3 ± 11.3	0.137
Loss of posterior height (X-rays – CT)	1.9 ± 8.6	-1.6 ± 8.6	2.7 ± 8.5	0.111

Supplementary Table S4. Univariate and multivariate logistic regression analyses for the prediction of metachronous fractures in patients with vertebral fractures located at the thoracolumbar junction, including the variables that showed statistically significant differences in the bivariate analyses. CM, Conservative management. OR, odds ratio. cOR, crude OR. aOR: adjusted OR. 95%CI, 95% confidence interval. U P-value of the univariate regression analysis. M P-value of the multivariate regression analysis. *Significant p-value. ^ For this variable, apart from sex and age, adjustment for the two variables that showed statistically significant differences in the bivariate descriptive analyses of tables 1 and 2 was made.

Variable	cOR [95%CI]	P-value[U]	aOR [95%CI]	P-value[M]
Treatment [Ref: CM]	0.253 [0.063-1.010]	0.052	0.120 [0.023-0.623]^	0.012
DGOU [Ref: OF2]	7.667 [2.094-28.068]	0.002	8.427 [2.133-33.293]	0.002
Intravertebral cleft	5.000 [1.376-18.167]	0.014	5.694 [1.459-22.228]	0.012
Loss of posterior height (CT)	1.097 [1.009-1.193]	0.029	1.108 [1.011-1.216]	0.029

Supplementary Table S5. Receiver-operating characteristics curve analysis for the variables of the multivariate model to predict the development of new fractures. The combined model includes the variables “treatment”, DGOU classification and loss of posterior height. AUC, area under the curve. 95%CI, 95% confidence interval.

Variable	AUC [95%CI]	P-value
Treatment [Ref: CM]	0.652 [0.522 – 0.782]	0.044
DGOU [Ref: OF2]	0.712 [0.569-0.854]	0.001
Intravertebral cleft	0.690 [0.552 – 0.829]	0.011
Loss of posterior height (CT)	0.717 [0.578-0.856]	0.013
Combined model	0.831 [0.704 – 0.957]	<0.001

Supplementary Figure S1. ROC curves of the univariate and multivariate models obtained for the prediction of metachronous vertebral fractures in patients with fractures located at the thoracolumbar junction. The colored curves represent the ROC curves for each significant independent variable based on the univariate logistic regression results (see legend). The blue curve refers to the multivariate model with 3 variables. The gray diagonal line corresponds to the reference of a random classification (line of no discrimination).

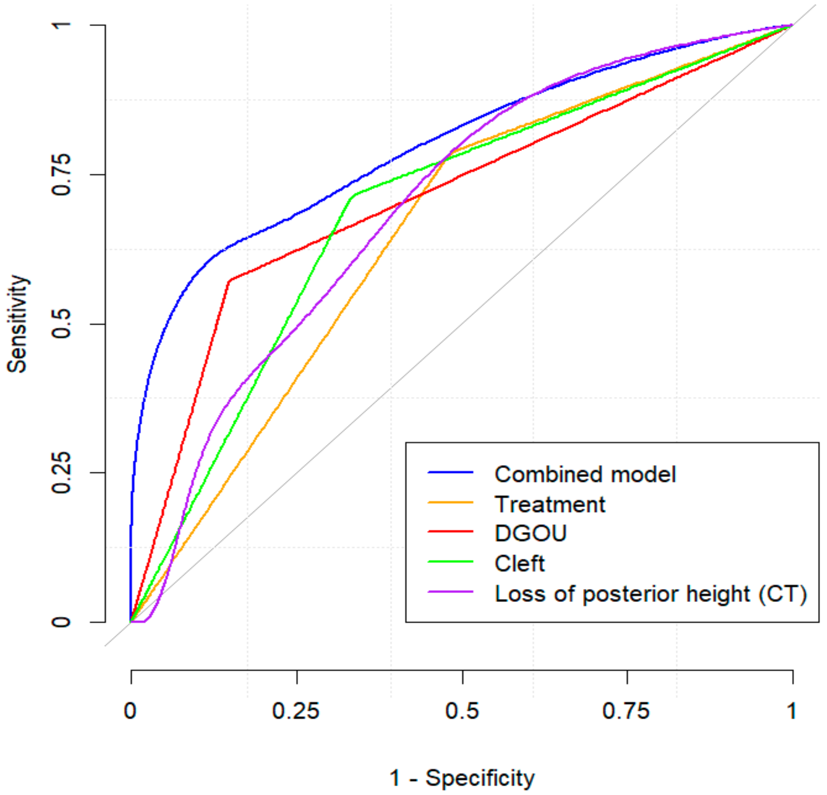

Supplementary Figure S2. Illustrative example of a patient without metachronous fractures in our sample. **A.** Initial X-rays. **B**. Initial CT (MPR set at 1 mm). **C.** Initial CT (MPR set at 100 mm for easier comparison with measurements on X-rays). **D.** Follow-up X-rays 2 years after diagnosis. This is an 86 year-old woman with a wedge, grade 1 (Genant's classification), OF2 fracture (DGOU classification) involving the upper endplate of L5 with intravertebral cleft (white arrow) in D). The difference in loss of PVH between X-rays and CT was 22.5%. She was managed with vertebroplasty (white arrow in D) and developed no metachronous fractures on follow-up.

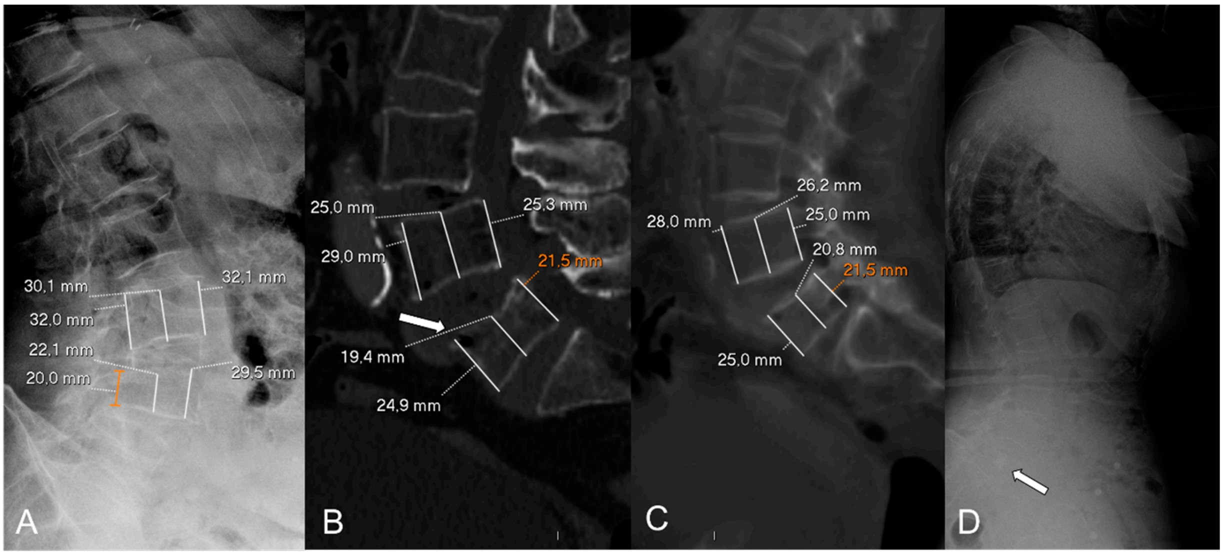

Supplementary Figure S3. Illustrative example of a patient who developed a metachronous fracture in our sample. A. Initial X-rays. B. Initial CT (MPR set at 1 mm). C. Initial CT (MPR set at 100 mm for easier comparison with measurements on X-rays). D. Follow-up X-rays. This is a 78 year-old woman with a wedge, grade 1 (Genant's numerical), OF2 (DGOU) fracture involving the upper endplate of L4. The difference in loss of posterior vertebral height between X-rays and CT was -3.5%. She was managed conservatively and developed L4 collapse and an L2 fracture at 3-year follow-up.

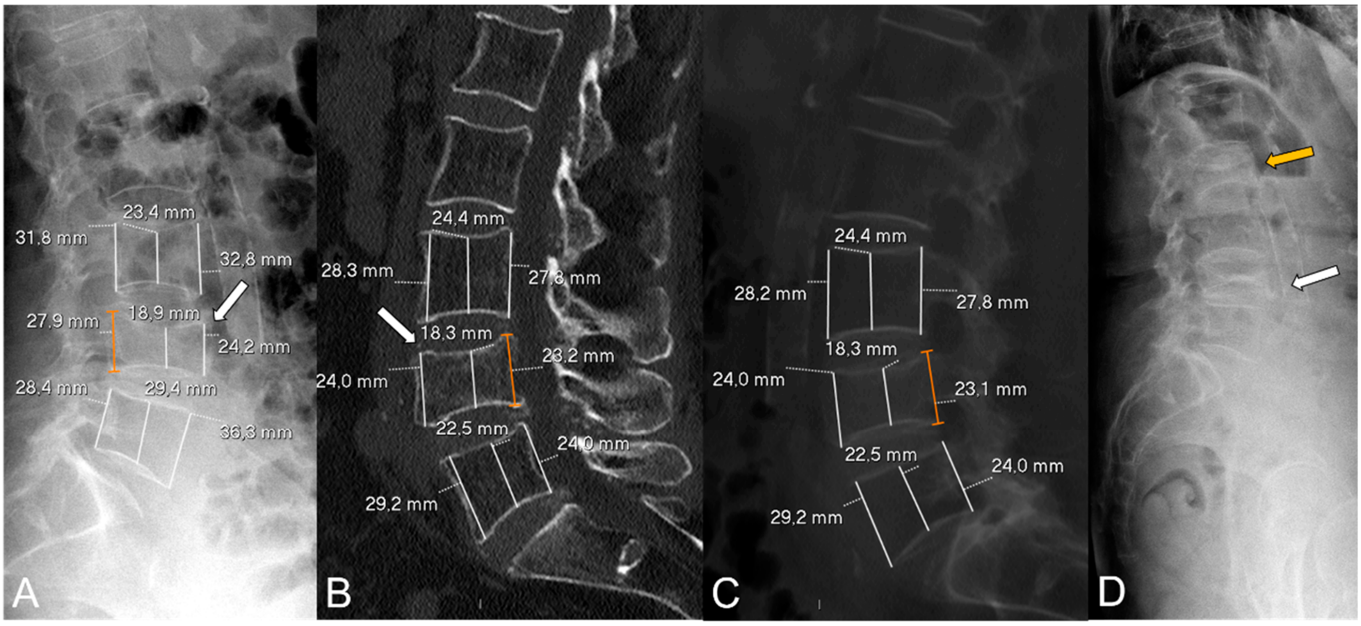

Supplement: Supplementary file 1 [file diagnostics-15-00160-s001.zip › diagnostics-3346275-supplementary.pdf]
